# RNA-Seq Comparison of Larval and Adult Malpighian Tubules of the Yellow Fever Mosquito *Aedes aegypti* Reveals Life Stage-Specific Changes in Renal Function

**DOI:** 10.3389/fphys.2017.00283

**Published:** 2017-05-09

**Authors:** Yiyi Li, Peter M. Piermarini, Carlos J. Esquivel, Hannah E. Drumm, Faye D. Schilkey, Immo A. Hansen

**Affiliations:** ^1^Department of Biology, New Mexico State UniversityLas Cruces, NM, USA; ^2^Department of Computer Science, New Mexico State UniversityLas Cruces, NM, USA; ^3^Department of Entomology, Ohio Agricultural Research and Development Center, The Ohio State UniversityWooster, OH, USA; ^4^National Center for Genome ResourcesSanta Fe, NM, USA; ^5^Institute of Applied Biosciences, New Mexico State UniversityLas Cruces, NM, USA

**Keywords:** mosquito, *Aedes aegypti*, Malpighian tubules, RNAseq, diuresis, detoxification

## Abstract

**Introduction:** The life history of *Aedes aegypti* presents diverse challenges to its diuretic system. During the larval and pupal life stages mosquitoes are aquatic. With the emergence of the adult they become terrestrial. This shifts the organism within minutes from an aquatic environment to a terrestrial environment where dehydration has to be avoided. In addition, female mosquitoes take large blood meals, which present an entirely new set of challenges to salt and water homeostasis.

**Methods:** To determine differences in gene expression associated with these different life stages, we performed an RNA-seq analysis of the main diuretic tissue in *A. aegypti*, the Malpighian tubules. We compared transcript abundance in 4th instar larvae to that of adult females and analyzed the data with a focus on transcripts that encode proteins potentially involved in diuresis, like water and solute channels as well as ion transporters. We compared our results against the model of potassium- and sodium chloride excretion in the Malpighian tubules proposed by Hine et al. (2014), which involves at least eight ion transporters and a proton-pump.

**Results:** We found 3,421 of a total number of 17,478 (19.6%) unique transcripts with a *P* < 0.05 and at least a 2.5 fold change in expression levels between the two groups. We identified two novel transporter genes that are highly expressed in the adult Malpighian tubules, which have not previously been part of the transport model in this species and may play important roles in diuresis. We also identified candidates for hypothesized sodium and chloride channels. Detoxification genes were generally higher expressed in larvae.

**Significance:** This study represents the first comparison of Malpighian tubule transcriptomes between larval and adult *A. aegypti* mosquitoes, highlighting key differences in their renal systems that arise as they transform from an aquatic filter-feeding larval stage to a terrestrial, blood-feeding adult stage.

## Introduction

### Aedes aegypti

Since the abandonment of the wide-spread vector control programs in the late 1960's, the yellow fever mosquito, *Aedes aegypti*, has rapidly re-emerged across the globe. The current range of this important disease vector puts ~2.5 billion people at risk for dengue fever, with an estimated 50–100 million cases per year. *A. aegypti* is also the primary vector of the viruses which cause yellow fever, chikungunya, and Zika (Nene et al., 2007; CDC, 2009; WHO, 2011; Rodriguez-Morales, 2015; Benelli and Mehlhorn, 2016; Lazear et al., 2016).

### Osmoregulation in *A. aegypti*

Larval and adult mosquitoes inhabit very different environments with diverse requirements regarding osmoregulation and excretion. During the larval (and pupal) life stages, mosquitoes are aquatic organisms that are immersed in a hypotonic environment (Bradley, 1987; Marusalin et al., 2012). Post-eclosion, adult *A. aegypti* are terrestrial organisms that face the threat of rapid dehydration. Accordingly, the osmoregulatory system must change rapidly for the mosquito to acclimate to the new environment (Bradley, 1987; Piermarini, 2016). Adult females are presented with an additional challenge when feeding on vertebrate blood. Within minutes, a female *A. aegypti* can ingest more than her own body weight in blood which drastically reduces her mobility (Clements, 1992). In order to regain mobility and maintain osmotic balance, *A. aegypti* females excrete at least 40% of the volume contained in a blood meal within 1–2 h (Williams et al., 1983; Drake et al., 2010). Vertebrate blood is composed of ~85% water, and a mixture of ions, sugars, and proteins (Scanlon and Tina, 2007). Thus, ingesting large volumes of blood affects osmotic balance and leads to the release and production of toxic metabolic wastes (e.g., heme, NH_3_) during digestion.

### Malpighian tubules

The Malpighian tubules (MT) are the key excretory tissues in mosquitoes for osmotic balance and excretion of wastes, such as xenobiotics and excess nitrogen. In *A. aegypti*, there are five MT that attach to the alimentary canal at the midgut/hindgut junction. The tubules are comprised of two main cell types (principal cells and stellate cells) that form a single epithelial layer around the lumen (Beyenbach and Piermarini, 2011). Stellate cells intercalate between principal cells along the distal segment of the tubule. Principal and stellate cells are thought to work in conjunction to maintain proper osmotic balance within the hemolymph (Beyenbach et al., 2010; Beyenbach and Piermarini, 2011).

### Mechanisms of transepithelial fluid secretion

In the current model of transepithelial fluid secretion in the MT of *A. aegypti*, a proton motive force is created by the principal cells through a V-type H^+^ ATPase (V-ATPase) located in the apical membrane (Beyenbach et al., 2010; Beyenbach and Piermarini, 2011; Piermarini, 2016). The V-ATPase pumps protons into the lumen creating proton and voltage gradients to power other electrogenic exchange transporters and ion channels that move Na^+^ and K^+^ from the hemolymph into the lumen of the tubules. Only some of the molecular mechanisms have been identified and characterized so far. The stellate cells exchange intracellular HCO_3_ with Cl^−^ from the hemolymph, then the Cl^−^ is transported to the lumen, presumably by chloride channels in stellate cells and/or a K,Cl cotransporter (KCC) in principal cells (Beyenbach, 2003; Beyenbach et al., 2010; Beyenbach and Piermarini, 2011; Piermarini et al., 2011). It is unknown exactly how water moves from the hemolymph to the lumen, however in *A. aegypti*, several aquaporin (AQP) mRNAs are enriched in the MT (Pietrantonio et al., 2000; Drake et al., 2010, 2015). In MT of *Anopheles gambiae*, Prip (AQP2 in *A. aegypti*) immunoreactivity has been located on the basolateral membrane of stellate cells in the distal segment and principal cells of the proximal segment (Liu et al., 2011; Tsujimoto et al., 2013). RNAi-mediated knockdown of selected AQPs in adult female *A. aegypti* resulted in greatly reduced whole-mosquito diuresis capabilities (Drake et al., 2010), consistent with roles in transepithelial water transport in the MT. Mosquito diuresis is regulated by a number of neuropeptides including kinins, cardioacceleratory peptides (CAPs), adiokinetic hormones (AKHs), and corticotropin-releasing factor related (CRF related) hormones (Gade, 2004).

### Excretion and detoxification

In addition to maintaining osmotic balance, the MT have a role in detoxification and excretion of metabolic waste products. Uric acid is thought to be transported into portions of the MT where it accumulates into crystals and assists with transport of water into the tubules by contributing to the osmotic gradient (O'Donnell et al., 1983). Enrichment of cytochrome p450's and glutathione *S*-transferases (GST) in the MT of *Drosophila, A. gambiae*, and *Aedes albopictus* indicates that this organ may also play a role in detoxification (Beyenbach et al., 2010; Ingham et al., 2014; Esquivel et al., 2016).

### This paper

Previous studies have characterized the transcriptome of the MT in *A. albopictus* (before and after blood feeding) (Esquivel et al., 2014, 2016) and *A. gambiae* (larval, adult, before, and after blood feeding) (Overend et al., 2015), but transcriptomic studies in the MT of *A. aegypti* have not previously been performed. The goal of this study was to characterize the changes occurring in the MT of *A. aegypti* mosquitoes when switching from an aquatic to a terrestrial life. We conducted an in depth RNA-seq analysis of MT from 4th instar larvae and adult females 3 days post-eclosion.

We found a high number of transcripts differentially expressed in the MTs of adult mosquitoes and larvae. We also identified potential sodium and chloride channels that expand our current model of diuresis in mosquitoes.

## Materials and methods

### Mosquito culture

Mosquitoes from the *A. aegypti* Rockefeller (ROCK) strain were raised as previously described (Price et al., 2011) with the exception that they were fed as larvae solely on cat food (Special Kitty Original, Wal-Mart stores, Bentonville, AR).

### Mosquito dissection and RNA isolation

MT were isolated from ~30 adult female or 30 larval (4th instar) *A. aegypti* in PBS and placed in Trizol® (Thermo Fisher Science). This was done in duplicate, creating two replicates for each group. Total RNA was then extracted from these tubule samples according to the manufacturers protocol (Chomczynski, 1993). A Nanodrop 1000 (Thermo scientific) was used to quantify total RNA concentration. RNA quality was assessed visually by an RNA gel.

### Illumina library preparation

Four micrograms of total RNA (the recommended maximum) from each sample was used to prepare a cDNA library for each sample using the TruSeq RNA Sample Preparation Kit v2 (Illumina), according to the manufacturer's protocol for low-throughput sample preparation, with the following modifications (Tsujimoto et al., 2017). Differences in the protocol and our preparation procedure were: (1) using PCR strip tubes instead of PCR plates, (2) Elute, Prime, Fragment mix was thawed on ice and mixed into each well of the RBP plate on ice, and (3) Ligation mix was thawed on ice and mixed into each well of the ALP plate on ice.

The resulting libraries were quantified using a Nanodrop 1000 and Agilent Bioanalyzer 2100 (Agilent Technologies, Santa Clara, CA) at New Mexico State University, then sent for sequencing to the National Center for Genome Resources (Santa Fe, NM). The sequencing lab further analyzed the libraries and pooled them for sequencing on a single lane of Illumina HiSeq2000 1 × 100 bp reads.

### Bioinformatics

Illumina reads were aligned using bowtie2 (v2.2.9) and Tophat2 (v2.1.1) to the *A. aegypti* reference transcripts available from Vectorbase (downloaded May. 5, 2016) (Lawson et al., 2009; Langmead and Salzberg, 2012). Each library was aligned separately. Alignments in each library for each transcript were tallied from bowtie2/Tophat2 results by htseq-count (Anders et al., 2015), then expression was compared between larvae and adults using the DESeq package in R (Anders and Huber, 2010). After alignment by bowtie2 and Tophat2, the FPKM (Fragments Per Kilobase of transcript per Million mapped reads) values and expression comparison of transcript isoforms were also calculated using Cuffdiff (Trapnell et al., 2010). A Pearson correlation test (Galton, 1877; Pearson, 1895) was used to compare DESeq results and Cuffdiff results with a correlation value of 0.98 and a *P*-value of lower than 2.2e-16.

### Functional clustering analysis of transcripts up-/down-regulated

For the clustering analysis, we followed a similar protocol to Esquivel et al. (2014, 2016). In brief, transcripts were separated based on their expression in the MT of adults relative to MT of larvae (i.e., down-regulated or up-regulated in adult MT). Then, transcripts were submitted to a standalone BLASTn (version 2.2.31) analysis against the *A. gambiae* transcriptome (PEST strain transcript sequences, AgampP4.4 geneset, v1.00; https://www.vectorbase.org/). The “best hit ortholog” for each *A. aegypti* transcript was retrieved. *A. gambiae* orthologs were subjected to a Database for Annotation, Visualization, and Integrated Discovery (DAVID, version 6.7) functional clustering analysis (Niaid, 2006). Only those functional clusters with an enrichment value >1.3 (corresponding to *P* < 0.05) were retrieved and recorded for further analysis.

## Results and discussion

### General sequencing results

**Figure 1:**
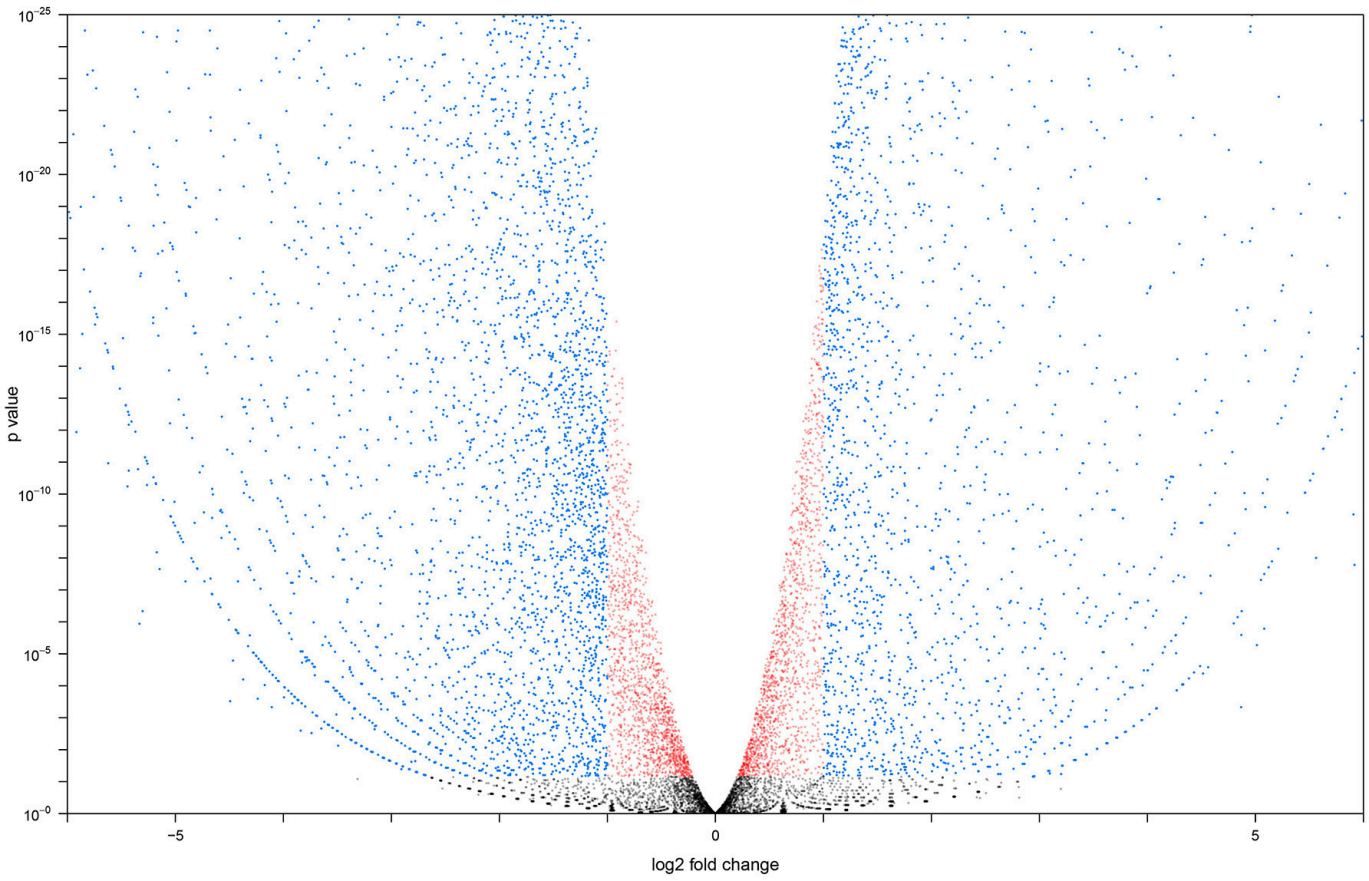
**Volcano plot generated with larval vs. adult transcript data**. Transcripts altered at a less than log 2 fold level are in red, transcripts altered at a greater than log 2 fold level, but with a *P* < 0.05 are in blue. Transcripts with an expression level altered more than log 2 fold and have a *P* > 0.05 are in black.

Two cDNA libraries for each group were sequenced, resulting in 26.3 and 26.2 million reads respectively for the adult MT libraries. All raw sequence data generated were submitted to the sequence read archive (Leinonen et al., 2011) and accepted under accession number SRX1884160 for larvae and SRX539939 for adult. Sequencing of larval libraries resulted in 31.0 and 31.2 million reads. Seventy-three to seventy-six percent of the reads in each library successfully aligned to the *A. aegypti* reference transcriptome. We calculated the Pearson correlation coefficient for our sample repeats. Coefficients between adult: adult and larval: larval libraries were 0.996 and 0.991, respectively. Our replicates exhibited a high degree of similarity, meaning that the transcriptome of *A. aegypti* Malpighian tubules was stable across biological repeats and not highly variable (Bonizzoni et al., 2011). The Pearson correlation between adult and larval libraries was 0.732, indicating potential changes in transcript expression between larval and adult MT. Figure 1 shows a volcano plot visualizing the differences in gene expression between the larval and adult transcriptomes. The overall sequence results are shown in Table S1.

### General comparison of larval and adult transcriptomes

Due to the vastly different osmoregulatory requirements of aquatic larvae and terrestrial adults, we expected to see significant differences between the transcript expression in their MT.

The pathway analysis performed using DAVID v6.7 (Huang et al., 2009) showed a total of 23 functional clusters among the transcripts up-regulated in adult MT, and 10 clusters among the transcripts down-regulated in adult MT (Table 1). Clusters were then manually categorized based on their putative biological role: transcription and translation (9 clusters), cell signaling and voltage-gated channels (10 clusters), protein sorting and trafficking (5 clusters), organelle sorting and trafficking (7 clusters), cell signaling (1 cluster), and redox and detoxification (1 cluster; Table 1).

**Table 1:** **Categorization of the enriched functional clusters of transcripts in the MT of larval and adult mosquitoes**.

**Biological pathway**	**Enrichment value**
**UP-REGULATED TRANSCRIPTS (IN ADULTS)**	
**Transcription and Translation**	
Protein amino acid phosphorylation	7.05
Ribosome	6.92
Transcription regulator activity	1.65
Bromodomain	1.44
Appendage morphogenesis	2.11
Regulation of cell development	2.06
Protein amino acid phosphorylation	3.43
Protein folding	2.34
Protein kinase, C-terminal	1.83
**Cell Signaling and Voltage-Gated Channels**	
Immunoglobulin domain	4.19
Voltage-gated channel activity	4.19
Src homology-3 domain	3.41
Transmembrane	2.6
Ligand-gated ion channel activity	2.39
Calcium channel activity	2.25
Tyrosine protein kinase	1.82
Pleckstrin homology-type	1.72
Voltage-gated potassium channel activity	1.63
Low density lipoprotein-receptor	1.4
**Protein Sorting and Trafficking**	
Ankyrin	1.67
dDENN	1.65
Microtubule-based process	1.56
GTPase regulator activity	1.53
**DOWN-REGULATED TRANSCRIPTS (IN ADULTS)**	
**Organelle Sorting and Trafficking**	
Cytoskeletal protein binding	3.02
Non-membrane-bounded organelle	2.25
Myosin complex	1.78
GTP binding	1.74
FERM central domain	1.65
Regulation of organelle organization	1.4
Actin cytoskeleton organization	1.33
**Cell Signaling**	
Calcium-binding EF-hand	2.07
**Protein Sorting and Trafficking**	
Cellular component morphogenesis	1.42
**Redox and Detoxification**	
ABC transporter-like	1.54

#### Functional clusters from up-regulated transcripts in adult MT

Inspection of the “transcription and translation” and “protein sorting and trafficking” categories suggest that MT of adult *A. aegypti* have enhanced activities related with (1) transcription of genes, (2) synthesis of proteins, (3) post-translational modification of proteins, and import/export of proteins among cellular organelles. In addition, the “cell signaling and voltage-gated channels” category gather a significant number of transcripts associated with active movement of ions (e.g., Na^+^, K^+^) through membranes for osmotic cellular regulation and/or cell signaling.

#### Functional clusters from down-regulated transcripts in adult MT

The category “organelle sorting and trafficking” and “protein sorting and trafficking” contains several transcripts associated with movement/re-arrangement of organelles by proteins (e.g., tubulin and myosin), suggesting that MT of non-blood fed females have reduced internal trafficking of cellular components. Furthermore, MT of adults are also showing a down-regulation of transcripts associated with cell signaling and redox/detoxification processes. These changes might correspond to the switch from an aquatic stage living in a relatively closed environment (i.e., a container) where toxins and wastes can accumulate and cannot be avoided until after pupal metamorphosis to the non-blood fed terrestrial/aerial stage living in a relatively open environment, where potential toxins and wastes can be avoided.

#### Most highly expressed transcripts in both samples

Tables 2, 3 show the top twenty most highly expressed genes in larval and adult MTs, respectively. Five transcripts are found in both tables:

- AAEL018662: predicted to encode cytochrome c oxidase subunit I
- AAEL004851: unknown protein
- AAEL017413: unknown protein
- AAEL018672: no protein (tRNA)
- AAEL017096: unknown protein.

**Table 2:** **Most abundant transcripts by read count in larvae**.

**Transcript ID**	**FPKM of adult**	**FPKM of larvae**	**Fold change a/l**	**Description**	**Stat**	***P*-values**
AAEL018672	9,254	60,002	0.15	No protein	−6.25	0.00
AAEL010789	37	40,445	0.00	Hypothetical protein	−43.37	0.00
AAEL001107	2,312	30,576	0.08	Hypothetical protein	−14.67	0.00
AAEL012645	5	27,327	0.00	Hypothetical protein	−43.77	0.00
AAEL017051	21	23,711	0.00	Trypsin-like cysteine/serine peptidase domain	−20.67	0.00
AAEL005849	3,050	18,649	0.16	Synaptic vesicle protein	−8.30	0.00
AAEL017413	5,081	16,268	0.31	Unknown protein	−6.93	0.00
AAEL017090	2	14,502	0.00	Unknown protein	−26.68	0.00
AAEL018662	7,982	12,573	0.63	Cytochrome c oxidase subunit I	−3.05	0.00
AAEL013777	2	11,037	0.00	Hypothetical protein	−32.21	0.00
AAEL017119	2,941	10,761	0.27	Unknown protein	−7.59	0.00
AAEL010675	2,281	10,496	0.22	Hypothetical protein	−8.49	0.00
AAEL002612	22	10,365	0.00	Hypothetical protein	−36.44	0.00
AAEL011557	44	10,324	0.00	Metalloproteinase, putative	−33.02	0.00
AAEL018673	1,778	10,149	0.18	Unknown protein	−4.54	0.00
AAEL004851	9,765	9,948	0.98	Hypothetical protein	−0.10	0.89
AAEL018674	982	8,736	0.11	Unknown protein	−4.59	0.00
AAEL004522	1,309	8,632	0.15	Hypothetical protein	−10.46	0.00
AAEL017096	4,359	8,128	0.54	Unknown protein	−4.14	0.00
AAEL002631	1	7,889	0.00	Hypothetical protein	−24.38	0.00

*The top 20 most highly expressed transcripts in A. aegypti larval MTs with adult expression levels for comparison purposes. Generated by Cuffdiff*.

**Table 3:** **Most abundant transcripts by read count in adult females**.

**Transcript ID**	**FPKM of adult**	**FPKM of larvae**	**Fold change a/l**	**Description**	**Stat**	***P*-values**
AAEL004851	9,765	9,948	0.98	Hypothetical protein	−0.10	0.89
AAEL018672	9,254	60,002	0.15	No protein	−6.25	0.00
AAEL018662	7,982	12,573	0.63	Cytochrome c oxidase subunit I	−3.05	0.00
AAEL018689	6,419	0	Inf	No protein	0.00	1.00
AAEL017198	6,275	4,880	1.29	Unknown Protein	1.47	0.04
AAEL007771	6,066	4,709	1.29	60S ribosomal protein L22	1.52	0.03
AAEL007824	5,821	5,011	1.16	Ribosomal protein S29, putative	0.86	0.22
AAEL017413	5,081	16,268	0.31	Unknown Protein	−6.93	0.00
AAEL018669	5,039	5,904	0.85	Cytochrome c oxidase subunit III	−0.99	0.16
AAEL004503	4,516	3,215	1.40	Hypothetical protein	1.90	0.01
AAEL017096	4,359	8,128	0.54	Unknown protein	−4.14	0.00
AAEL005451	4,046	4,599	0.88	Hypothetical protein	−0.74	0.29
AAEL016995	4,027	5,965	0.68	Unknown protein	−2.64	0.00
AAEL004151	3,928	3,981	0.99	Hypothetical protein	−0.08	0.91
AAEL017231	3,896	6,017	0.65	Unknown protein	−2.41	0.00
AAEL011656	3,875	3,893	1.00	40S ribosomal protein S15	−0.03	0.97
AAEL017491	3,757	0	Inf	Unknown protein	NA	0.00
AAEL009403	3,723	2,987	1.25	Hypothetical protein	1.28	0.07
AAEL018498	3,721	3,905	0.95	Unknown protein	−0.10	0.89
AAEL003942	3,659	3,014	1.21	60S ribosomal protein L44 L41, putative	1.12	0.11

*The top 20 most highly expressed transcripts in A. aegypti adult MTs with larval expression levels for comparison purposes. Generated by Cuffdiff. 60S ribosomal proteins and 40S ribosomal proteins are increased in adults*.

In larvae, cysteine/serine peptidase domain, synaptic vesicle protein, cytochrome c oxidase subunit I, metalloproteinase, putative, and 16 hypothetical proteins round out the top 20.

In the adult MTs, ribosomal proteins make up 4 of the 20 most highly expressed transcripts, while in larvae there are no ribosomal proteins in the twenty mostly highly expressed transcripts.

#### Most highly changed transcripts between larva and adult

Table 4 shows the top 10 most upregulated genes in larvae compared to adult while Table 5 shows the top 10 most upregulated genes in adults compared to larvae. Four of 10 genes that are highly upregulated in the larval stage are annotated as hypothetical proteins while seven of the 10 adult genes are annotated. In adult MT, ribosomal genes were expressed at very high levels and exhibited very high degrees of upregulation compared to larvae.

**Table 4:** **Most altered transcripts in larvae**.

**Transcript ID**	**No. of reads Adult**	**No. of reads Larvae**	**DEseq fold change l/a**	**FPKM of adult**	**FPKM of larvae**	**Description**	**Stat**	***P*-values**
AAEL004745	0	4,323	1.19E+04	0.00	427.80	Pupal cuticle protein, putative	−11.74	0.00
AAEL011504	0	3,416	9.65E+03	0.00	735.75	Pupal cuticle protein, putative	−11.36	0.00
AAEL008451	13	72,532	9.43E+03	0.33	3,388.57	Alpha-amylase	−45.76	0.00
AAEL013773	4.5	21,608	7.49E+03	0.23	1,960.42	Hypothetical protein	−28.37	0.00
AAEL005340	10.5	46,415	7.40E+03	0.42	3,261.52	Hypothetical protein	−40.62	0.00
AAEL017056	4	17,764	6.84E+03	0.35	2,690.37	Peptidoglycan recognition protein sc2	−26.73	0.00
AAEL013777	28	103,708	6.43E+03	1.61	11,037.10	Hypothetical protein	−60.12	0.00
AAEL011930	5.5	21,337	6.20E+03	0.30	1,984.13	Hypothetical protein	−30.15	0.00
AAEL007044	1	5,528	6.14E+03	0.03	279.69	Lipase	−16.15	0.00
AAEL008045	6.5	24,312	6.07E+03	0.11	682.97	Hexamerin 2 beta	−32.27	0.00

*The most upregulated transcripts in larval vs. adult MTs. Generated by DEseq. Several proteins with chitin binding domains are highly increased in larvae*.

**Table 5:** **Most altered transcripts in adult females**.

**Transcript ID**	**No. of reads Adult**	**No. of reads Larvae**	**DEseq fold change a/l**	**FPKM of adult**	**FPKM of larvae**	**Description**	**Stat**	***P*-values**
AAEL000776	4,891	1	2,634	108	0	Hypothetical protein	12.04	0.00
AAEL001703	1,4524	3	2,298	617	0	Serine-type enodpeptidase, putative	20.50	0.00
AAEL004386	2,1634	5	2,191	306	0	Peroxinectin	25.34	0.00
AAEL002422	2,718	6	2,110	190	0	Cytoplasmic polyadenylation element binding protein (cpeb)	26.26	0.00
AAEL003404	22,096	6	2,052	316	0	Hypothetical protein	26.13	0.00
AAEL004390	27,478	8	1,913	393	0	Peroxinectin	29.26	0.00
AAEL014516	1,758	0	1,901	87	0	Metalloproteinase, putative	8.65	0.00
AAEL006281	1,377	0	1,530	31	0	Glucose transporter (sugar transporter)	8.34	0.00
AAEL007657	3,871	1	1,445	22	0	Low-density lipoprotein receptor (ldl)	13.07	0.00
AAEL009504	1,199	0	1,355	58	0	Hypothetical protein	8.18	0.00

*The most upregulated transcripts in adult vs. larval MTs. Generated by Deseq*.

Based upon these results (data presented in Tables 1–5), the adult MTs appear to be more heavily geared toward protein synthesis than those of larvae, which is consistent with results from the MT of non-blood fed *A. albopictus* (Esquivel et al., 2016).

### Ion and water transport machinery

**Figure 2:**
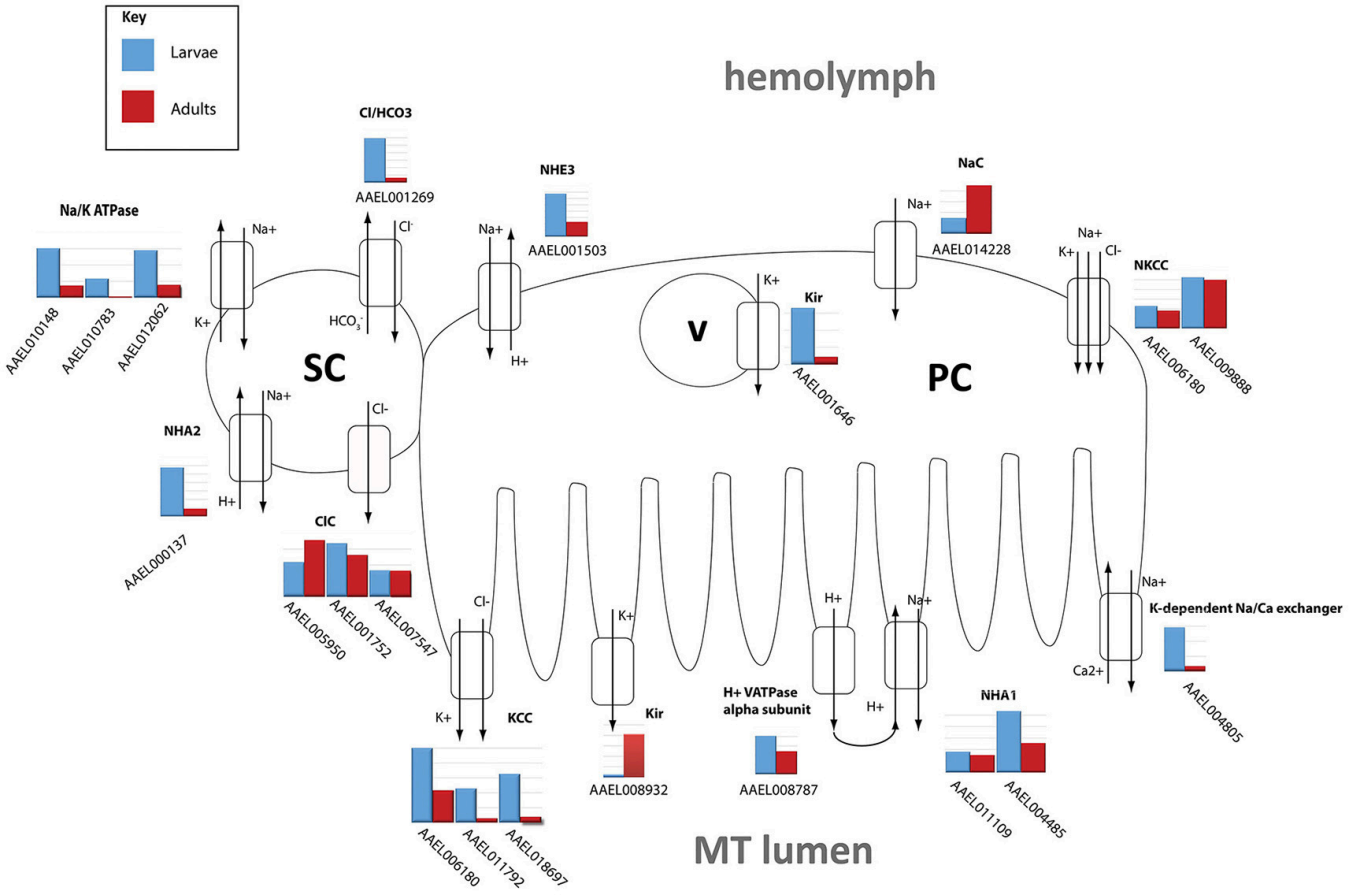
**Model of NaCl and KCl excretion**. Expression levels of transporters hypothesized to be involved in excretion of NaCl and KCl in *Aedes aegypti*. Shown is the log scale transcript expression of each transporter we found to be expressed, larvae in red, adults in blue. The cellular localization of several of these transporters has not been determined and is therefore hypothetical. PC, principal cell; SC, stellate cell; v, vesicle.

The DAVID functional cluster analysis identified four groups associated with ion transport: “voltage-gated channel activity,” “transmembrane,” “ligand-gated ion channel activity,” and “voltage-gated potassium channel activity” (Table 1). Other transcripts associated with epithelial ion transport are also found within the “cell signaling and voltage-gated channels” clusters (Table 1). In addition, a manual search was performed to find transcripts associated with the current model of transepithelial fluid secretion in MT (Hine et al., 2014; Piermarini, 2016). We were able to identify at least one member of each gene family represented in this model in both larval as well as adult MT transcriptomes. Figure 2 shows the relative expression levels of some model-associated transcripts. This model includes sodium/potassium ATPases (Na/K ATPase), a chloride/bicarbonate anion exchanger (Cl/HCO_3_), sodium/proton antiporters (NHA1, NHA2), a sodium/proton exchanger (NHE3), inward rectifier potassium channels (Kir1 & 3), a sodium channel (NaC), sodium/potassium/chloride cotransporters (NKCC), chloride channels (ClC), potassium/chloride cotransporters (KCC), and V-ATPase subunits. Our analysis resulted in the identification of several novel paralogous transcripts expressed in the MT and also allowed the identification of some genes that are dominantly expressed. We found that many transcripts show life stage-dependent expression. Most transcripts that we analyzed had a greater level of expression in the larval MTs with only a few exceptions. For example, the putative sodium channel (NaC) AAEL014228, the only one of three NaC genes that is highly expressed in MTs, is 3.1 fold increased in adult relative to larval MTs, suggesting that it may play a role in the post-prandial diuresis of females.

We found that Kir1 (AAEL008932) was up-regulated in adults while Kir3 (AAEL001646) was up-regulated in larvae which confirms qPCR results published earlier (Yang et al., 2017). In adults, Kir1 is expressed on the basolateral membrane of stellate cells where it contributes to most of the transepithelial secretion of K^+^ (Piermarini et al., 2015). On the other hand, Kir3 is expressed in intracellular compartments of principal and stellate cells in adult MT, but its physiological role is unknown (Piermarini et al., 2015).

NHA2 (AAEL000137) was highly up-regulated in larvae (6.46x), consistent with a putative role in the absorption of luminal Na^+^ as proposed by Xiang et al. (2012).

The NKCC transcripts AAEL009888 and AAEL006180 were not differentially expressed.

Catalytic subunit A of V-ATPase (AAEL008787) was 2.96x higher in larvae, while many other subunits show moderate up-regulation in larvae (see Table 6). This is in contrast to microarray data from *A. gambiae* which indicates that in this mosquito species V-ATPase subunits are generally higher expressed in adults compared to larvae (Overend et al., 2015).

**Table 6:** **V-ATPase subunit expression**.

**Transcript ID**	**No. of reads Adult**	**No. of reads Larvae**	**DEseq fold change a/l**	**FPKM of adult**	**FPKM of larvae**	**Description**	**Stat**	***P*-values**
AAEL014053	3,505	999	1.97	43.13	21.80	Vacuolar proton ATPases	9.03	0.00
AAEL010819	1,1978	8,139	0.83	465.16	556.55	Vacuolar ATP synthase subunit H	−2.54	0.02
AAEL002464	9,193	6,257	0.83	808.64	968.44	Vacuolar ATP synthase subunit f	−2.40	0.02
AAEL012819	8,516	7,524	0.64	406.57	631.73	Vacuolar ATP synthase subunit g	−5.93	0.00
AAEL006390	13,712	13,725	0.56	182.11	320.95	Vacuolar proton ATPases	−7.41	0.00
AAEL003743	4,374	4,601	0.53	45.75	84.31	Vacuolar proton ATPases	−9.55	0.00
AAEL015594	659	714	0.52	30.35	57.77	Vacuolar ATP synthase subunit c	−6.58	0.00
AAEL005173	3,178	3,642	0.49	40.07	80.34	Vacuolar ATP synthase subunit c	−9.26	0.00
AAEL012113	6,108	7,219	0.48	400.88	849.73	Vacuolar ATP synthase proteolipid subunit	−9.60	0.00
AAEL006516	11,805	14,645	0.45	192.42	420.00	Vacuolar ATP synthase subunit h	−10.75	0.00
AAEL012035	34,113	51,513	0.37	386.50	1, 027.63	Vacuolar ATP synthase subunit e	−14.26	0.00
AAEL011025	15,032	23,751	0.36	325.64	904.92	Vacuolar ATP synthase subunit ac39	−14.93	0.00
AAEL005798	27,446	44,250	0.35	225.54	640.38	ATP synthase subunit beta vacuolar	−15.35	0.00
AAEL008787	24,374	40,642	0.34	268.44	787.98	V-type proton ATPase catalytic subunit A	−15.58	0.00
AAEL000291	75,297	164,326	0.26	850.71	3, 281.70	V-type proton ATPase 16 kDa proteolipid subunit	−19.49	0.00

*Comparison of V-ATPase subunit expression in larval vs. adult MTs by Deseq*.

These results suggest there may be potential differences in transport rates of certain ions between larval and adult MT, due not only to changes in expression levels but also of transporter types which may have different kinetics.

As mentioned earlier, we found a transcript encoding the alpha-subunit of the sodium/potassium ATPase (AAEL008787) to be 2.96x more abundant in MTs of larvae vs. adults (Figure 2). The Na/K-ATPase has recently been considered to play an important role in transepithelial fluid secretion by MT of *A. aegypti* (Hine et al., 2014). Immunoreactivity for the alpha subunit localizes to the basolateral membranes of stellate cells in the distal segment and principal cells in the proximal segment (Patrick et al., 2006), and ouabain significantly inhibits K^+^, Cl^−^, and fluid secretion in isolated MTs (Hine et al., 2014). Thus, our finding is consistent with a potentially important role of this ATPase in transepithelial fluid and cation secretion by adult MTs.

The larval challenge is to retain NaCl and KCl and to remain hypertonic by excreting a dilute urine, in the face of a constant hypotonic external force. In contrast, the adult female mosquito is challenged with the sudden need to excrete a large amount of fluid following a blood meal. The larvae need to get rid of water constantly and retain ions, whereas the adult female needs to retain water to prevent dehydration until the moment she takes a blood meal. At that point she needs to get rid of water and ions very quickly (Patrick et al., 2001; Beyenbach, 2003; Beyenbach and Piermarini, 2011). Thus, the transcriptome of adult female MTs is likely primed with molecular mechanisms to fulfill her diuretic needs in response to a blood meal.

The model hypothesizes the existence of a basolateral sodium channel (NaC), which allows the entry of Na^+^ into the principal cells from the hemolymph, and an apical chloride channel, which allows the movement of Cl^−^ from the stellate cells to the tubule lumen (Figure 2). In our analysis, we found one NaC (AAEL014228), and two ClC's (AAEL005950, AAEL001752) to be highly expressed in adult tubules (Figure 2). These two ClCs are orthologous to ClC-a and ClC-b in *Drosophila*, respectively. ClC-a (2x increase) and b (1.1x increase) were found to be more highly expressed in adults. ClC-a is known in *Drosophila* to be exclusively expressed in stellate cells and responds to diuretic hormone-induced secretion, while ClC-b is a housekeeping channel (Cabrero et al., 2014). There are three ClC-type channels in *Drosophila*, whereas in *A. aegypti* there are seven. The large expansion of genes in *A. aegypti* is potentially due to differences in selective pressure as a result of different feeding habits of fruit flies vs. mosquitoes (Wang et al., 2004).

Notably, a potassium-dependent sodium-calcium exchanger (NCKX1) was very highly expressed in larval and adult MT (AAEL004805). An ortholog of this transcript was also highly abundant in the MT of adult female *A. albopictus* and *A. gambiae* (Overend et al., 2015; Esquivel et al., 2016). NCKX1 may be associated with the response of MTs to kinins. That is, after stimulation with kinin peptides, there is an increase of intracellular Ca^+2^, which stimulates the secretion of Cl^−^ (Yu and Beyenbach, 2002). A basolateral Na^+^/Ca^2+^ exchanger could provide a mechanism for lowering intracellular [Ca^2+^] after the influx of Ca^2+^ or maintaining low intracellular [Ca^2+^] before activation by kinins.

These previously unstudied transporters in *A. aegypti* represent potential mechanisms for disrupting diuresis, and exploiting as targets for novel insecticides development (Raphemot et al., 2013).

It is still unclear how the MT transport water from the hemolymph to the tubule lumen, but paracellular transport through septate junctions and transcellular transport through aquaporins are the most likely routes. RNAi studies by our group suggest that aquaporins play a major role (Drake et al., 2010, 2012; Benoit et al., 2014). In the present study, we found reads aligned against all six currently known aquaporins in the *A. aegypti* genome, as well as the B and C isoforms of aquaporin 5 (Eglp2) in both samples (Table 7). The pattern of expression observed for each aquaporin in the MT was very similar to the results of Drake et al. with aquaporins 1(Drip), 2 (Prip), 4 (Eglp1), and 5 (Eglp2) being the most highly expressed, while 3 (Bib) and 6 (Aqp12L) are expressed at very low levels (Drake et al., 2010). We found that aquaporins are generally upregulated in larvae with the exception of AQP6. However, our statistical analysis did not show significant changes except for AQP 1 and 3 (*P* < 0.05). These results may indicate that AQP expression overall is higher in MT of larvae vs. adults and that transcellular water transport is more important in MT of larvae than in those of adults.

**Table 7:** **Aquaporin expression**.

**Transcript ID**	**No. FPKM of Adult**	**No. FPKM of Larvae**	**DEseq fold change a/l**	**Description**	**Stat**	***P*-values**
AAEL014255-RA (AQP6)	0.78	0.45	1.74	aquaporin, putative	1.29	0.10
AAEL003550-RA (AQP2)	237.51	218.72	1.09	aquaporin	0.29	0.69
AAEL005001-RA (AQP4)	279.96	287.96	0.97	aquaporin	−0.15	0.85
AAEL005008-RA (AQP5)	91.45	105.22	0.87	aquaporin	−0.70	0.36
AAEL005008-RC (AQP5)	11.66	14.04	0.83	aquaporin	−0.35	0.66
AAEL003512-RA (AQP1)	86.87	198.66	0.44	aquaporin-1	−4.61	0.00
AAEL014108-RA (AQP6)	0.03	0.10	0.29	aquaporin, putative	0.00	1.00
AAEL005008-RB (AQP5)	0.20	0.81	0.24	aquaporin	−0.42	0.62
AAEL004741-RA (AQP3)	1.24	5.50	0.22	aquaporin transporter	−7.69	0.00

*Comparison of aquaporin channel expression of different transcript isoforms in larval vs. adult MTs by Cuffdiff. In general, aquaporin expression is lower in adult vs. larval MTs*.

### Waste excretion and detoxification

In addition to maintaining fluid balance, the Malpighian tubules are a major site of detoxification and excretion of waste products, from pesticides to excess nitrogen and iron, through mechanisms such as cytochrome P450's and glutathione S-transferases (Folwell et al., 2006; Yang et al., 2007; Dow, 2009; Chahine and O'Donnell, 2011). We investigated which of these detoxification mechanisms are present by examining the expression of transcripts and comparing adult and larval expression.

#### Cytochrome P450 and glutathione

We found reads aligning to 181 identified cytochrome P450 transcripts and 115 of them were differentially expressed with statistical significance (*P* < 0.05); of these we found 35 of 115 significant transcripts to be expressed at low levels (<100 reads) (Table S2) in both larvae and adults. Of the transcripts expressed at a low level, we found 17 (all exhibiting at least a 2 fold induction) to be more highly expressed in adults compared to larvae and 18 (17 exhibiting at least a 2 fold induction) to be more highly expressed in the larvae compared to adults. The most highly expressed cytochrome P450 transcript in adults was AAEL004054 and in larvae AAEL017539. We compared the changes in expression to the expression profiles of cytochrome P450's found to be involved in xenobiotic metabolism and pyrethroid resistance (Poupardin et al., 2010; Bariami et al., 2012). We found that many cytochrome P450's that are up-regulated in resistant strains were upregulated in larvae. However, there were many exceptions and it does not appear that the complement of larval up-regulation carry over to adult expression.

There were reads aligning to 34 transcripts which have been categorized as glutathione-interacting enzymes and are likely to be involved in detoxification pathways (Table S3). Eighteen of these were expressed above a low level (both larvae and adult >1,000). 14 were found to have statistically significant differential expression, with 10 more abundant in larvae, and four more abundant in adults. The most highly expressed detoxification-related transcript in MT of larvae and adults was AAEL001071 (GSTD5). We found the epsilon class of GSTs, which are capable of detoxification of DDT, to be much more highly expressed in adults (2–1,500 fold higher) (Lumjuan et al., 2007, 2011).

Our results indicate that mosquito larvae have a much more active detoxification system compared to adults, which makes physiological sense, because larvae are more likely to encounter persistent exposure to harmful wastes and xenobiotics in closed aquatic larval habitats.

**Figure 3:**
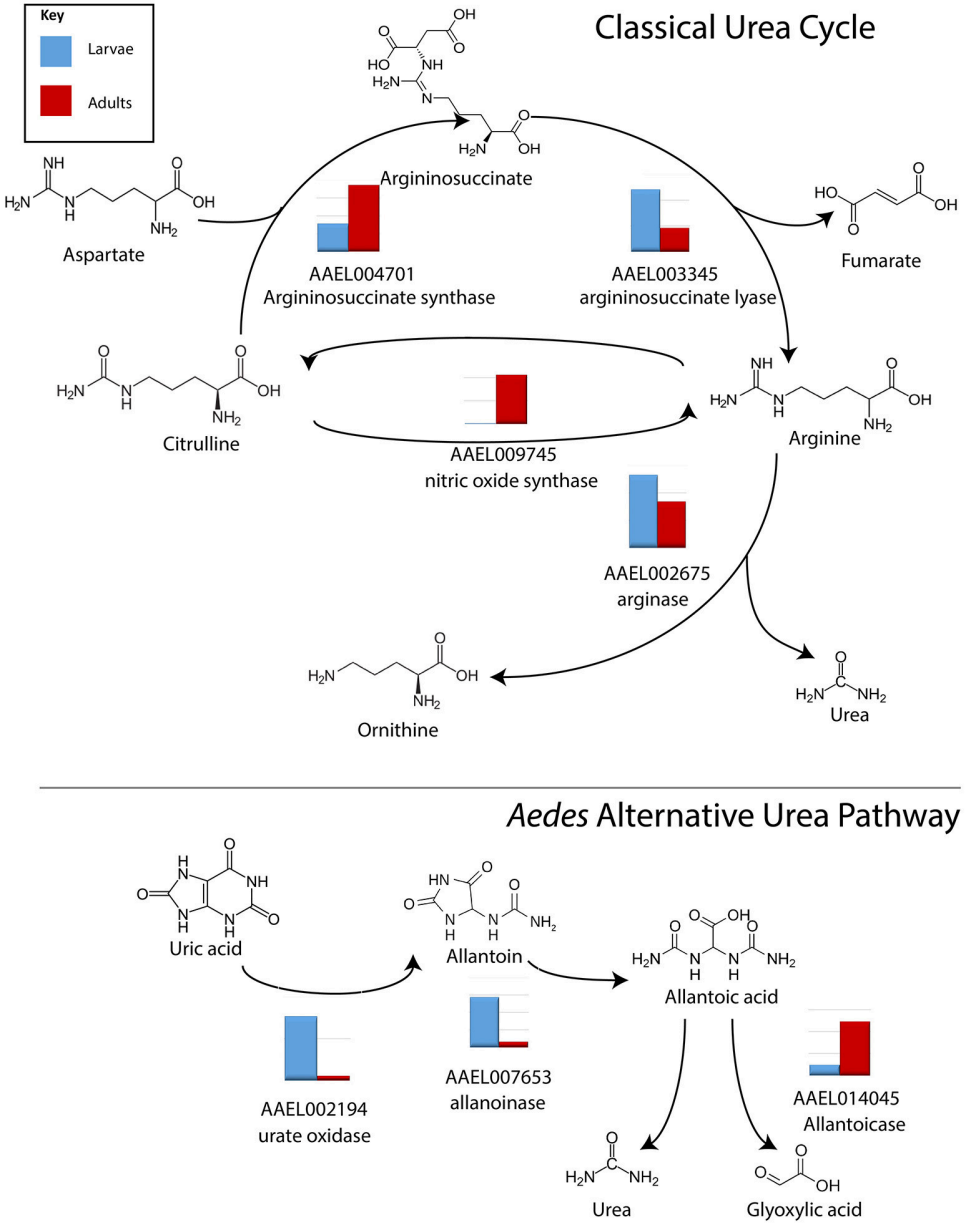
**Conversion of nitrogen in waste products**. Log scale expression of transcripts for enzymes involved in the synthesis of nitrogen waste products in *A. aegypti* MTs. In general, in the classical urea cycle, transcripts are lower in larvae compared to adults, with the exception of argininosuccinate lyase. In the alternative urea pathway, transcripts are higher in larvae compared to adults with the exception of allantoicase. Shown is the log scale transcript expression of each transporter we found to be expressed, larvae in red, adults in blue.

Three transcripts relating to uric acid production, aldehyde oxidase, xanthine dehydrogenase and uricase in particular, were found to be significantly more abundant in MT of larvae vs. adults, by 13.9, 7.6, and 7.5 fold, respectively (Table S4). Arginase, which produces urea, was found to be evenly expressed in larvae and adults (Isoe and Scaraffia, 2013). However, expression of transcripts encoding arginase in MT of both larvae and adults is relatively low (>1,000 reads) while other enzymes in the urea cycle are much more highly expressed, and significantly more abundant in larvae (Figure 3). Results indicate that adults may be producing more urea as a waste product, while larvae are producing uric acid; representing possible differential nitrogen excretion between adult females and larvae (Kuzhivelil and Mohamed, 1998; von Dungern and Briegel, 2001; Scaraffia et al., 2008; Isoe and Scaraffia, 2013). This could be due to an aquatic vs. terrestrial lifestyle or larvae consuming cat food with protein while the adults consume sugar water. However, we did not detect significant differences in the amount of uric acid between MT of adults (non-blood fed) and larvae (data not shown).

We found 8 of 13 ferritin transcripts, including a ferritin precursor, to be highly expressed within the MT (>1,000 in both adult and larval MT, Table S5). Overall, ferritins were more abundant in the larval MT (1.4x). However, we would expect this to be reverse after the adults blood feed and this seems to be the case according to data in aeGEPUCI (Dissanayake et al., 2010). That is, in whole mosquitoes, several ferritins expressed at a low level increase rapidly post-blood meal and some of the highly expressed transcripts increase.

From these results, it appears that MT of adults potentially utilize stored iron to create heme peroxidases for immunity, dealing with oxidative stress or for other uses prior to blood meal. Using some stored iron in this way could assist in dealing with the influx of iron following a blood meal.

## Conclusions

This study found significant changes between the MT transcriptomes of 4th instar larvae and adult female *A. aegypti* 3 days post-eclosion and identified several genes of potential interest for further functional analysis. Complete results of DEseq and Cuffdiff analysis are shown in Tables S6–S8.

Alterations in the expression of classical diuretic genes, such as ion and water transporters, reflect differing challenges in undertaking diuresis in aquatic and terrestrial environments, while changes in the abundance of glutathione S-transferases and cytochrome P450s likely reflect changes in the environment and diet. Changes in expression of enzymes of the nitrogen cycle and iron metabolism may reflect the dramatic change in diets between larval and adult mosquitoes.

We also have identified candidates for sodium and chloride channels predicted by the models of KCl and NaCl excretion of Hine et al. (2014).

## Author contributions

Performed the experiments: HD, FS, and YL; Analyzed the data: YL, PP, FS, and CE; Wrote the paper: IH and PP; Edited the manuscript: YL, PP, CE, FS, and IH.

## Funding

This project was supported by the NIH grants SC1AI109055 and P20GM103451, and pilot funds from the National Center for Genome Resources (NCGR). The funders had no role in study design, data collection and analysis, decision to publish, or preparation of the manuscript.

### Conflict of interest statement

The authors declare that the research was conducted in the absence of any commercial or financial relationships that could be construed as a potential conflict of interest.

## Supplementary material

Table S1 **Overall sequencing results**. General statistics pertaining to the sequencing run. Number of reads and % aligning is the number of reads from the library which aligned to the reference transcripts using Bowtie2 and Tophat2.

Table S2 **Transcripts related to Cytochrome P450**. Expression of transcripts related to Cytochrome P450 in adult and larval MTs.

Table S3 **Transcripts related to glutathione**. Expression of transcripts related to glutathione in adult and larval MTs.

Table S4 **Transcripts related to uric acid production**. Expression of transcripts related to uric acid production in adult and larval MTs.

Table S5 **Transcripts related to iron sequestration**. Expression of transcripts related to iron sequestration and metabolism in adult and larval MTs.

Table S6 **Completed DEseq result**. The completed DEseq result table with annotation for each gene.

Table S7 **Completed Cuffdiff Gene result**. The complete Cuffdiff result table of each gene.

Table S8 **Completed Cuffdiff isofrom result**. The complete Cuffdiff result table for different isoforms.
