# Corrigendum: RNA-Seq Comparison of Larval and Adult Malpighian Tubules of the Yellow Fever Mosquito *Aedes aegypti* Reveals Life Stage-Specific Changes in Renal Function

**DOI:** 10.3389/fphys.2017.00843

**Published:** 2017-11-07

**Authors:** Yiyi Li, Peter M. Piermarini, Carlos J. Esquivel, David P. Price, Hannah E. Drumm, Faye D. Schilkey, Immo A. Hansen

**Affiliations:** ^1^Department of Biology, New Mexico State University, Las Cruces, NM, United States; ^2^Department of Computer Science, New Mexico State University, Las Cruces, NM, United States; ^3^Department of Entomology, Ohio Agricultural Research and Development Center, The Ohio State University, Wooster, OH, United States; ^4^Molecular Biology Program, New Mexico State University, Las Cruces, NM, United States; ^5^National Center for Genome Resources, Santa Fe, NM, United States; ^6^Institute of Applied Biosciences, New Mexico State University, Las Cruces, NM, United States

**Keywords:** mosquito, *Aedes aegypti*, Malpighian tubules, RNAseq, diuresis, detoxification

The author David P. Price was not included as an author in the published article. The authors apologize for this error and state that this does not change the scientific conclusions of the article in any way.

The revised author list:

Yiyi Li^1, 2^, Peter M. Piermarini^3^, Carlos J. Esquivel^3^, David P. Price^4^, Hannah E. Drumm^1^, Faye D. Schilkey^5^, and Immo A. Hansen^1, 2, 4, 6^

^1^ Department of Biology, New Mexico State University, Las Cruces, NM, United States

^2^ Department of Computer Science, New Mexico State University, Las Cruces, NM, United States

^3^ Department of Entomology, Ohio Agricultural Research and Development Center, The Ohio State University, Wooster, OH, United States

^4^ Molecular Biology Program, New Mexico State University, Las Cruces, NM, United States

^5^ National Center for Genome Resources, Santa Fe, NM, United States

^6^ Institute of Applied Biosciences, New Mexico State University, Las Cruces, NM, United States

## Author contributions

Performed the experiments: DP, HD, FS, and YL; Analyzed the data: DP, YL, PP, FS, and CE; Wrote the paper: IH, PP, FS, CE, DP; Edited the manuscript: YL, PP, CE, FS, and IH.

### Conflict of interest statement

The author declares that the research was conducted in the absence of any commercial or financial relationships that could be construed as a potential conflict of interest.

